# On the Availability of Certain Antiseptics in the Prophylactic Treatment of the Oral Cavity

**Published:** 1885-08

**Authors:** W. D. Miller

**Affiliations:** Berlin


### ON THE AVAILABILITY OF CERTAIN ANTISEPTICS IN THE PROPHYLACTIC TREATMENT OF THE ORAL CAVITY.

BY PROF. W. D. MILLER, BERLIN.

In the INDEPENDENT PRACTITIONER of 1884, page 283, I published a table giving the results of a series of experiments on the antiseptic action of a considerable number of agents used in the treatment of the oral cavity. A few new materials have since been added to the number, and I here reproduce the complete table, in order to institute a comparison between the relative powers of the materials experimented upon:

| ANTISEPTIC AGENTS. | DEVELOPMENT OF FUNGI PREVENTED. |
|---|---|
| Bichloride of mercury | 1-100,000 |
| Nitrate of silver | 1-50,000 |
| Peroxide of hydrogen* | 1-8.000 |
| Iodine | 1-6,000 |
| Iodoform | 1-5,000 |
| Naphthalin | 1-4,000 |
| Salicylic acid ( Crystals ) * | 1-2,000 |
| Oil of Mustard | 1-2,000 |
| Benzoic acid* | 1-1,500 |
| Permanganate of potash | 1-1,000 |
| Eucalyptus | 1-600 |
| Carbolic acid | 1-500 |
| Hydrochloric acid | 1-500 |
| Biborate of soda* | 1-350 |
| Arsenious acid * | 1-250 |
| Chloride of zinc | 1-250 |
| Lactic acid | 1-125 |
| Carbonate of sodium | 1-100 |
| Listerine* | 1-20 |
| Alcohol | 1-10 |
| Chlorate of potash* | 1-8 |

It must, however, be apparent to every one, that the real value of these antiseptics should not be put down as proportional to the numbers in the above table. To say that the bichloride of mercury is, for dental uses, two hundred times as efficient as carbolic acid, would be as inadmissible as to say that dynamite is, in proportion to its greater power, more valuable for industrial purposes than are horses. The question of the availability of different forms of energy, which has long been one of greatest interest to physicists, merits also, in the study of antiseptics, a thorough consideration. A partial solution of this question I have endeavored to present in the following notes :

The substances thus far experimented upon are bichloride of mercury 1-2,500 and 1-5,000; carbolic acid 1-100; permanganate of potash 1-4,000; peroxide of hydrogen 1-10; boracic acid 1-50; benzoic acid 1-100 and 1-200; salicylic acid 1-100 and 1-200; listerine undiluted ; oil of peppermint and oil of wintergreen in agreeable strength for a mouth-wash. I have endeavored to take, in

* The substances indicated by a star were tested upon pure cultures of the fungus described in Vol. V, page 341, Fig. 2 ; the others on mixtures. In a general way stronger solutions are necessary to sterilize mixtures than to sterilize pure cultures.

each case, the highest concentrations which may be used in the mouth, either as a wash or on the brush. The one per cent solution of benzoic and salicylic acids (in twenty per cent. alcohol) is rather too sharp to be ordinarily used for rinsing the mouth, but may be applied on the brush; the same is the case with listerine.

Since in rinsing the mouth one does not usually keep the liquid in contact with the teeth for more than a minute, we need as an antiseptic mouth-wash a substance which is capable of devitalizing the fungi of the mouth in one minute, or less. The following method may be employed in making this determination: We procure;—1st, a tube containing a pure culture of some one of the more important fungi of the human mouth; we may call this the infecting tube. 2d. A tube containing 0,5 cc. of the antiseptic to be experimented with; this we may call the antiseptic tube. 3d. A number of tubes containing 5,0 cc. of culture liquid; we will call these the culture tubes. A small drop or bead is conveyed from the infecting tube to the antiseptic tube on a loop of fine platinum wire, and then, at intervals varying from one-quarter of a minute to fifteen minutes, beads are conveyed from the antiseptic tube to each of the culture tubes in succession (one bead to each tube). These tubes are then kept at the temperature of the oral cavity. If the fungi were devitalized by the action of the antiseptic, the culture liquid remains clear; otherwise, it becomes cloudy in a period varying from seven to sixty hours.

These experiments should be doubly controlled:

1st. Two culture tubes should be infected, using only sterilized water in the antiseptic tube. These two tubes should become cloudy in seven to ten hours, and if others of the tubes become cloudy in the same time, it indicates that the antiseptic through which the fungi were passed had no effect upon them. The retardation in the appearance of the cloudiness will give us a measure of the action of the antiseptic in those cases where it effected only a partial sterilization.

2d. The experiment should be repeated, using only sterilized water in both the infecting and antiseptic tubes. In this case the culture tubes should remain permanently clear. Should the culture tubes, however, become cloudy, it would indicate clumsy work on the part of the experimenter, and his experiments would be absolutely worthless. It is advisable to sterilize the air of the room with vaporized sublimate before performing these experiments.

The results will be seen in the adjoined table. In the first column are given the antiseptics, and in the second the time of exposure necessary to a complete devitalization:

| | | |
|---|---|---|
| Salicylic acid | 1–100 | ¼ minute.* |
| Benzoic acid | 1–100 | ¼ " |
| Listerine, pure | | ¼ to ½ min. |
| Salicylic acid | 1–200 | ½ " |
| Benzoic acid | 1–200 | 1 to 2 " |
| Bichloride of mercury | 1–2,500 | ½ to ¾ " |
| " " " | 1–5,000 | 2 to 5 minutes. |
| Eucalyptus | 1–200 | 5 to 10 " |
| Peroxide of hydrogen, 10 per cent solution | | 10 to 15 " |
| Carbolic acid, 1 per cent | | 10 to 15 " |
| Oil of peppermint | | 10 to 15 " |
| Permanganate of potash | 1–4,000 | over 15 " |
| Boracic acid | 1–50 | " 15 " |
| Oil of wintergreen | | " 15 " |

It is seen at a glance that of the substances tested in this series, only four are available for the prophylactic treatment of the oral cavity, particularly of the teeth. These are bichloride of mercury, benzoic acid, salicylic acid and listerine.

Of these four, I think the bichloride is without doubt the most effective, because its action continues longer. In the attempt to sterilize the oral cavity, a very serious difficulty is encountered in the fact that the antiseptic cannot, in many cases, be brought into contact with all portions of it; the free surfaces may become sterilized, but approximal surfaces, fissures, cavities, especially when stopped up with food and various deposits, escape the action of the antiseptic, unless it may be kept for some minutes in the mouth. It is here that the bichloride possesses an advantage; after the body of the liquid has been put out of the mouth, the traces remaining continue their action even after they have been diluted forty times by the oral secretions. It is also much more penetrating than the less soluble benzoic and salicylic acids. Any one may convince himself of the effectiveness of corrosive sublimate by a very simple experiment. Rinse the mouth thoroughly with a solution (1–2,500), keeping it one minute in the mouth. Half an hour later, by chew-

* Probably less time is necessary, but the transference could not well be made more quickly.

ing a small lump of sugar, secrete about 3 to 5 cc. of saliva, bring it into a sterilized test-tube, and keep it at blood temperature. It will be found, after twenty-four hours, that no fermentation has followed, though it usually shows itself in three to four hours.

Unfortunately, the bichloride of mercury possesses one great disadvantage in its highly poisonous character. It seems, however, scarcely possible that any harm could result from its use in so dilute a form. The maximum dose of corrosive sublimate, *pro die*, is 0,1 and if we suppose that each time the mouth is washed (once daily) 0,5 ccm. of the solution gets into the system (a very high estimate), then it would be five hundred days before the amount which may be administered in one day would be reached. As remarked above, one can scarcely believe that any bad effect could be possible, and yet the element of certainty is lacking, and though I use the solution myself constantly, even as strong as 1 to 1,000, I hesitate to recommend it to my patients. Considering the great interest which this question must have for every one, I would strongly urge members of the profession to test this matter upon themselves and report the results. In no other way can we satisfactorily solve the question. For self-experimentation I would recommend a solution of 1 to 1,000, with sufficient wintergreen to disguise the taste. It has, furthermore, been claimed that a solution of bichloride of mercury has power to decalcify the teeth. This, however, needs confirmation.

As to salicylic acid, it is claimed by some that it is injurious to the teeth; others deny that it has any effect of that kind. Buch (*Journal of Mat. Med.*, *April*, 1880), after using a solution of salicylic acid in strength of 3 to 1,000 for some weeks, was obliged to discontinue its use, because he noticed "a curious feeling in his mouth; the teeth became softer and their surfaces rough through the formation of the salicylate of lime." On the other hand, a chemist in Berlin has used a much stronger solution for over ten years. His teeth, when he began, were in a most deplorable state, but in a short time he succeeded in arresting the progress of caries, of which there is at present not a trace in his mouth, while the old cavities are brown and hard. No evidence whatever of any action upon the teeth can be seen. It seems, however, pretty well established that salicylic acid is a substance which must be used in the mouth with great caution, to say the least.

Benzoic acid might be substituted for salicylic, provided it should not also turn out to have an injurious effect on the teeth.

Particularly noteworthy is the rapidity with which listerine acts upon the fungi; it appears to be one of the strongest and safest of the available antiseptic solutions. It owes its antiseptic property, in all probability, more to the boro-benzoic acid which it contains than to the eucalyptus oil.

In comparing the two tables given above, we notice what appear at first to be very great contradictions. Why does the peroxide of hydrogen, which in one series stands very high, fall so low in the other? and why should listerine, which is forty times weaker than the peroxide, kill the fungi thirty times quicker? I can account for this striking difference only on the supposition that the rapidity with which a given antiseptic acts is by no means necessarily proportional to its strength. Perhaps an imperfect analogy may be seen in the action of poisons upon the animal body, some of which produce almost instantaneous death, while others, equally fatal, act only after the lapse of a certain time. It is necessary, first of all, that the antiseptic pass through the cell membrane before it can act upon the cell contents. This passage takes place very quickly in case of some, very slowly in case of others, although the latter, once through, may act more powerfully than the former. We must also bear in mind that the proportions of the first table prevent only the development of the fungi, while in the second their devitalization is accomplished. There is a very great difference between the two. Many substances and conditions prevent, by their mere presence, the development of fungi, without in the least rendering them incapable of development when they are brought into the proper medium. We need, of course, for most dental uses, materials which produce a complete devitalization.

It is, furthermore, an important fact that the substance which is to be sterilized may so act upon the antiseptic as to materially lessen or completely destroy its power. In this respect the permanganate of potash is very sensitive. Again, an interesting question which has often come up in the course of the experiments is this: Is the strength of a given antiseptic proportionate to its concentration? i. e., is a two per cent. solution just twice as efficient as a one per cent. solution? My experience would teach me that this is not at all necessarily the case.

The most unexpected results were obtained with the peroxide of hydrogen. I hoped to find in it a substance which, as a mouth-wash, would far surpass anything at present available, but was very seriously disappointed, as the results were anything but satisfactory. It makes, however, a very *agreeable* wash, and I shall not myself give up its use until I have tested it on other fungi. Solutions of this substance are liable to deteriorate. One which I now have was as strong when fresh, in the proportion of 1 to 12,000, as it now is in the strength of 1 to 8,000.

